# Detection of human papillomavirus in epithelial lesions of the conjunctiva

**DOI:** 10.1590/S1516-31802000000500003

**Published:** 2000-09-01

**Authors:** Maristela Amaral Palazzi, Clélia Maria Erwenne, Luísa Lina Villa

**Keywords:** Human papillomavirus, Polymerase chain reaction, Epithelial lesions, Conjunctiva, Papilomavírus humano, Reação de polimerização em cadeia, Lesões epiteliais, Conjuntiva

## Abstract

**CONTEXT:**

Many factors like exposure to UV radiation, climatic conditions, genetic predisposition, immunological state and, more recently, the presence of HPV have been implicated in the genesis of some lesions of the conjunctiva, especially the carcinoma.

**OBJECTIVE:**

To evaluate the presence of HPV DNA in acquired lesions of the conjunctiva and in normal mucosa.

**TYPE OF STUDY:**

Cross-sectional study.

**SETTING:**

A public university referral center (the Ophthalmology Service of the A.C. Camargo Hospital - A. Prudente Foundation, São Paulo).

**PARTICIPANTS:**

Thirty patients with acquired lesions of the conjunctiva and 60 matched controls (by age and sex) were evaluated in this study, from June 1993 to March 1995.

**PROCEDURES:**

The detection of HPV DNA in the normal conjunctiva and in acquired lesions was done by the PCR technique and dot blot hybridization. The material was collected by scraping the normal mucosa and the surface of the lesions. A fragment of fresh frozen tissue and paraffin embedded specimens of each lesion were also included.

**MAIN MEASUREMENTS:**

The association between the HPV infection and the presence or absence of conjunctival lesions.

**RESULTS:**

Sequences of HPV DNA were detected in 4 of the 31 lesions evaluated (12.9%) and in the healthy mucosa of one individual of the control group (1.6%). HPV type 16 was detected in 2 carcinomas and in the normal mucosa of one individual of the control group. HPV type 11 was demonstrated in 2 papillomas of one patient with lesions in both eyes.

**CONCLUSIONS:**

The low frequency of HPV DNA found in the lesions of this sample and the detection of the viral genome in the normal mucosa indicate that there is a weak possibility of association between HPV infection and the carcinoma of the conjunctiva.

## RESUMO

**CONTEXTO:**

Vários fatores como exposição à radiação ultravioleta, condições climáticas, predisposição genética, estado imunológico e, mais recentemente, a presença do papilomavírus humano têm sido implicados na etiopatogênese das lesões neoplásicas da conjuntiva, em especial dos carcinomas.

**OBJETIVO:**

Avaliar a presença de DNA do HPV em uma série de lesões adquiridas da conjuntiva e na mucosa clinicamente normal.

**TIPO DE ESTUDO:**

Estudo transversal.

**LOCAL:**

Centro de referência universitário (Serviço de Oftalmologia do Hospital A. C. Camargo - Fundação Antônio Prudente).

**PARTICIPANTES:**

Trinta pacientes com lesões epiteliais adquiridas da conjuntiva e 60 controles pareados por sexo e idade foram incluídos no estudo, no período de Junho de 1993 a Março de 1995.

**PROCEDIMENTOS:**

A pesquisa de DNA do HPV, na conjuntiva normal e nas lesões adquiridas, procedeu-se através das técnicas de reação de polimerização em cadeia (PCR) e hibridização em pontos. O material para estudo foi coletado por esfregaço da mucosa normal e da superfície das lesões conjuntivais. Os fragmentos congelados da peça cirúrgica e os espécimes incluídos em parafina de cada uma das lesões foram igualmente incluídos na investigação.

**VARIÁVEIS ESTUDADAS:**

A associação entre a infecção pelo HPV e a presença ou não de lesões conjuntivais.

**RESULTADOS:**

Seqüências do DNA do HPV foram detectadas em 4 de 31 lesões avaliadas (12,9%) e na mucosa clinicamente sadia de um indivíduo do grupo controle (1,6%) (P = 0,07). O HPV tipo 16 foi detectado em dois carcinomas invasivos e na mucosa clinicamente normal de um indivíduo do grupo controle. O HPV tipo 11 foi identificado em dois papilomas de um único paciente portador de lesões em ambos os olhos.

**CONCLUSÕES:**

A baixa freqüência do DNA do HPV, encontrada nas lesões desta amostra, e a detecção do genoma viral na mucosa clinicamente sadia indicam que há uma fraca possibilidade de associação entre a infecção pelo HPV e os carcinomas da conjuntiva.

## INTRODUCTION

Human papillomavirus (HPV) is a DNA virus from the Papovaviridae family that has been identified in a variety of epithelial lesions of the skin and mucosa. Up to the present day, more than 70 types of HPV have been isolated, some with known oncogenic potential infecting the genital tract, urinary bladder, larynx, oral cavity and conjunctiva.^1^ The potential for malignant transformation of some HPV is related to its ability to bind tumor suppressor proteins in the host, thus affecting the cellular cycle of the infected cells.^2^ The HPV already identified in the conjunctiva are of the high risk types 16 and 18 and the low risk types 6 and 11.^1,3^

The first demonstration of HPV in the conjunctiva was in the 1980's, from the identification of the capsid antigen in papillomas by employing immunohistochemistry.^4,5,6^ In subsequent years, authors in Europe and U.S. identified and typed HPV DNA in papillomas, dysplasias and carcinomas of the conjunctiva and also in normal mucosa, using techniques of molecular hybridization.^3, 7-15^

The prevalence of some HPV is universal for some lesions and for some specific sites like the genital tract. However, the frequency of HPV DNA detection in the conjunctiva varies considerably.^1^

We proposed to investigate the presence of HPV DNA in a sample of epithelial lesions of the conjunctiva, motivated by the scarcity of publications and the variability of the results relating to HPV findings in the conjunctiva.

## METHODS

A cross-sectional study was conducted in order to search for HPV DNA in epithelial lesions of the conjunctiva, in a sample of patients referred to the Ophthalmology Service of the Cancer Hospital - Antonio Prudente Foundation, São Paulo, from June 1993 to March 1995.

Three samples of each lesion were collected for analysis and included: swab, fresh frozen fragment of the surgical specimen and paraffin-embedded material. Swabs of the conjunctiva, apparently normal, of the opposite eye were also performed, as well as in sixty matched controls. The controls were matched by age (+/- 3 years) and sex.

*Swab.* After the instillation of anesthetic drops, a swab was performed over the lesion in the affected eyes and between the limbus and inferior fornix in the fellow eye using an appropriate spiral brush (Cytobrush, Medscand, Sweden). After the swab had been performed, the Cytobrush was immediately immersed in Tris solution (10mM, EDTA 1mM, pH 7.5). Samples were stored at 4^o^C until analysis.

*Fresh frozen fragment of the surgical specimen and paraffin-embedded material*. After removal, the lesions were sectioned using a surgical blade, from limbus to fornix or from limbus to palpebral canthus, over a rigid surface. About 1/3 of the specimen was put into a dry vial and sent for immediate freezing at −20^o^C. The other 2/3 were put into formaldehyde 10% and sent for histopatho-logical analysis by the Department of Pathology of the Cancer Hospital. The surgical specimens were evaluated by optical microscopy after staining with hematoxylin-eosin.

The DNA of the sample was isolated using ionic change columns in the “Glass Max DNA Isolation Spin Cartridge System” (Gibco / BRL, Gaithersburg, MD, USA). The DNA of the samples was submitted to PCR with consensus primers MY 9/11,^16^ which amplify 450 bp of L1 of several types of HPV, in order to verify the presence of HPV DNA. The primers G-73 and G-74, which amplify 268 of in the human *B* globin gene, were employed as internal controls of the integrity and suitability of the DNA in each sample. The typing of different HPV was done by dot blot hybridization using 28 specific-type probes,^16^ or through the digestion of the PCR products by restriction enzymes^2^ capable of identifying about 40 different types of HPV.

For the paraffin-embedded tissue the GP 5+ / 6+ primers were also employed, which amplify a fragment of 150 bp of the L1 gene of most genital HPVs.^17^

Positive controls included samples of genital tumor with known HPV DNA content, and negative controls included normal epithelial tissue, HPV-negative. Commercially available cervical-carcinoma cell lines (SiHa, HeLa cells) were used as additional controls.

### Statistical Methods

The Chi-square test was used to access the significance of the association between the HPV infection and the presence or absence of conjunctival lesions.

## RESULTS

Thirty-one lesions were obtained from thirty patients, with ages varying from 10 to 82 years (mean 49.7 years, median 54, SD 18.7). In the control group the age varied from 10 to 83 years (mean 49.7, median 54.5, SD 18.2). Seventeen cases (and 34 controls) were men and thirteen were women (26 controls), producing a proportion of 56.6% male to 43.3% female.

Twenty patients (66.7%) were white and 10 (33%) non-white. There were no Negro or Oriental patients in this sample. Twenty-nine patients (96.7%) had uni-lateral lesions and one (3.3%) had lesions in both eyes. The left eye was involved in 18 cases (58%). Fourteen lesions were new (45.1%), 15 recurrent (48.4%) and 2 were considered residuals (6.5%) from clinical evidence and histopathological proof.

All the lesions were located in the palpebral fissure. In 24 cases (77.4%) the lesions involved both the bulbar conjunctiva and the limbus, simultaneously.

Optical microscopy evaluation of hematoxylineosin stained sections identified: 10 invasive squamous cell carcinomas, 9 carcinomas in situ, 1 basal cell carcinoma of the eyelid infiltrating the conjunctiva, 3 dysplasias, 2 papillomas, 2 non-melanotic nevi and 4 inflammatory lesions. The last two were included in this sample because they corresponded to elevated lesions appeared just at the site of previously resected carcinomas (Table 1).

**Table 1:** Histopathological diagnosis of conjunctival lesions analyzed for the presence of HPV DNA, the frequency of its association and type of HPV found

Diagnosis	no. lesions	freq. HPV DNA	HPV type
ca “in situ”	9	0	--
invasive scc	10	2	16
bcc	1	0	--
severe dysplasia	2	0	--
mild dysplasia	1	0	--
papilloma	2	2	11
non-melanotic nevus	2	0	--
chronic inflammation	3	0	--
pyogenic granuloma	1	0	--
**Total (%)**	**31**	**4(12%)**	

Legend: ca = carcinoma; scc = squamous cell carcinoma; bcc = basal cell carcinoma of the eyelid infiltrating the conjunctiva.

Sequences of HPV DNA were detected in 4 of the 31 evaluated lesions (12.9%) and in the healthy mucosa of an individual in the control group (1.6%) (P = 0.07).

DNA of HPV type 11 was identified in 2 papillomas of one patient with lesions in both eyes. DNA of HPV 16 was detected in 2 invasive squamous cell carcinomas and in the swab of the apparently healthy conjunctiva in one person of the control group. A viral genome was detected and identified in the paraffin-embedded material of 4 lesions, in the swab of 3 lesions and in the frozen tissue of 2 out of 4 lesions. Table 2 shows the distribution of individuals with HPV conjunctival infection according to their identification characteristics, the histopathologic diagnosis and the type of HPV found.

**Table 2:** Distribution of patients and controls of the study with conjunctival infection by HPV, according to the age (years), sex, race, the histopathologic diagnosis of their conjunctival lesions and the type of HPV found

I.D.	Case 1	Case 16	Case 26	Control 10b
sex	M	F	M	M
age(years)	10	15	40	70
race	nw	nw	w	w
diagnosis	scc	papilloma	scc	no lesion
HPV type	16	11	16	16

Legend: nw = non-white; w = white; scc = squamous cell carcinoma.

## DISCUSSION

The frequency of HPV DNA detection in the conjunctiva varies considerably, as has been shown by some investigators over the last few years (Table 3).

**Table 3:** Distribution of studies concerning HPV in lesions of the conjunctiva, published between 1986 and 1994, according to the first author, the histopathologic diagnosis of the lesions, the frequency and type of HPV found and the technique employed

Reference	HPV type	frequency	diagnosis	technique
Naghashfar,^8^ 1986.	6	1/1	papilloma	SB / ISH
McDonnell,^6^ 1986.	16	15/23	papilloma	ISH
		0/28	dysplasia/ca	ISH
McDonnell,^9^ 1989.	16	12/16	dysplasia/ca	PCR
Lauer,^10^ 1990.	16,18	4/5	dysplasia/ca	PCR
Odrich,^11^ 1991.	16	3/3	dysplasia/ca	
McDonnell,^3^ 1992.	16	35/42	dysplasia/ca	PCR
		37/42		
Mincione,^12^ 1992.	6,11	2/4	papilloma	ISH
Tuppurainen,^13^ 1992.	6,11,16,18	0/4	carcinoma	PCR / ISH
Cha,^15^ 1994.	16	21/31	dysplasia/ca	PCR
	18	2/31		

Legend: SB = Southern blot; ISH = in situ hybridization; PCR = polymerase chain reaction.

In this sample, of the 29 types of HPV researched, including some that have not been numbered yet, only the HPV types 11 and 16 were detected. According to the literature these types of HPV are also the ones most frequently found in the conjunctiva.

We detected HPV DNA in 4 of the 31 lesions (12.9%) and in the apparently healthy conjunctiva of one individual of the control group (1.6%). The DNA of HPV 11 was detected in 2 papillomas (in one patient). The HPV 16 was found in 2 invasive carcinomas and in the conjunctiva of one individual of the control group. We did not detect HPV DNA in the fellow eye of the patients bearing conjunctival lesions.

The detection of HPV in the apparently healthy mucosa has previously been reported in the literature from different anatomical locations such as the cervix and larynx, as well as the conjunctiva.^3,18,19^

The latency of the virus can be demonstrated by the detection of HPV DNA in the mucosa in the absence of clinical lesions and subclinical changes.^20^ About 15 to 40% of women with positive HPV in the cervix do not have any kind of mucosal change.^21,22^

In the conjunctiva, investigators have identified HPV DNA 16 in swabs from clinically uninvolved eyes and have also reported persistent infection by HPV for many years after successful eradication of epithelial neoplasia, without any recurrences.^3^ The significance and the implications of virus latency in the conjunctiva, as in other mucosa, need additional investigation.

The modes of HPV transmission, depending on the HPV type and location, may involve casual physical contact, sexual contact and perinatal vertical transmission. The access of HPV to the conjunctiva is still an object of investigation. Transmission to the conjunctiva may occur as a result of fetal passage through an infected birth canal or by ocular contact with contaminated hands or objects.^23^ The former mechanism could easily explain the conjunctival papillomas found in children, but not necessarily those of adults. The presence of HPV 6 and 11 in adult conjunctival papillomas may reflect either activation of a latent HPV infection acquired as above, or an infection acquired later in life by transmission from other mucosal sites through either of the latter mechanisms.^8,22,24^

The detection of HPV types 6 and 11 in papilloma is very common, and these two types are probably responsible for the majority of the papillomas of the human conjunctiva, at least in children and young adults.^25^ On the other hand, the variations in the frequency of HPV detection for the types 16 and 18, in dysplasias and carcinomas, are significant.^1^

There are papillomas of viral origin and non-viral ones. Though they cannot be distinguished from a histopathological viewpoint, viral papilloma can be suspected if they occur in children or young adult, as they are generally pediculous, bilateral, multiple, located in the conjunctival fornices or in the eyelid and sometimes suffer spontaneous regression. The non-infectious papillomas occur in adults. They are diffuse or sessile, develop in the bulbar conjunctiva and according to some authors can represent a benign squamous hyperplasia or a dysplasia.^23^

The carcinomas and the precursor lesions, unlike the papillomas, do not present known characteristics that could give the suspicion of any association with HPV. The HPV seem to be associated with some, but not all carcinomas. Some authors have found that certain carcinomas, especially the verrucous types in the tonsil and larynx, are in their great majority associated with HPV, while other types do not seem to have viral etiology.^26^

In our study we did not observe any similarities among the lesions, nor even among the patients with HPV-positive lesions, that could put them in a homogenous group distinct from the other individuals and from the controls of the study.

The HPV-positive lesions were not different from the others in terms of their appearance, as the majority were presented as elevated vascularized gelatinous masses. Nor did the timing of their appearance distinguish them very well, with this being at about 7 months for HPV-positive lesions (variation: 1 to 12 months) and 9 months for the HPV-negative ones (variation: 1 to 48 months).

The recurrence of epithelial neoplasia of the conjunctiva is considered common.^27,28,29^ Some authors have detected a high frequency of association of recurrent lesions with some HPV.^15^ In spite of a significant proportion of recurrent lesions in our sample (48.4%), HPV was found in none of them. The HPV-positive lesions corresponded to the first conjunctival tumors presented by those patients at the time of our initial evaluation.

We decided to investigate the HPV DNA using 3 samples from each lesion as it is known that viral DNA can be found either superficially and diffusely dispersed in the epithelium, or concentrated in a focal way in a group of cells. The samples included: swab of the lesion, a frozen fragment of the surgical specimen and the paraffin-embedded material. HPV DNA was found in all the paraffin-embedded tissue from the positive lesions, in the swabs of 3 out of 4 lesions, and in the frozen tissue from 2 of them. Due to the observation of discordance in relation to HPV DNA positivity in different samples from the same lesion (positive biopsy, negative swab and vice versa), investigators have suggested the collection of material by either swab or biopsy, in order to obtain more conclusive results.^30,31^

The polymerase chain reaction or PCR has been widely used over recent years for the detection of HPV in cutaneous and mucosal lesions.^1^ We used the PCR technique in our study because it shows the most sensitivity of all the available methods. However, exactly because of its sensitivity, false-positive results coming from contamination are relatively common. Thus, rigorous control of the assay conditions is necessary.^32^ In order to avoid cross contamination among the samples, changes of materials like gloves and aerosol-free tips were routinely made at all steps of the assay.

Discrepancies in the prevalence of HPV detected by PCR may be related to different variables such as: primers used, reaction conditions, cycling parameters and magnesium concentration. Thus, all these parameters were rigorously observed and controlled in this study. Aspects inherent to the samples and to the method were also analyzed.

The amplification of the human globin gene, which is present in all cells, was applied to all biological samples and proved that the gene material was sufficient and adequate for the DNA study, thus excluding false-negative results.

We decided on an evaluation of the L1 gene, which is not lost at the time of virus integration into the host cell. Such deletion can potentially happen with the commonly used E1/E2 genes. The use of the latter could in some cases explain certain false-negative results.^11^

The frequency of the HPV found in epithelial lesions from our sample can be considered low, when we compare this to the findings of other studies.

Some evidence supports the hypothesis that the HPV does not act alone in the development of epithelial lesions of the conjunctiva, like findings of HPV DNA in dysplasias and carcinomas in such variable frequencies, its detection in apparently healthy mucosa, and its persistent positivity years after the removal of the lesions.

Exposure to excessive ultraviolet-B light (UV-B) has been identified by numerous previous studies as a major etiological factor in the development of ocular surface squamous neoplasia.^33^ It may be that ultraviolet light or other carcinogens act in concert with viral infection to transform cells.^9^ In fact, the role of HPV in the conjunctiva has yet to be proven. The close association of some human papillomavirus (HPV 16) with a majority of cervical carcinomas implies an important role for the virus in this type of cancer. However, in relation to the epithelial neoplasia of the conjunctiva, this can not be considered as a verisimilitude. At present, the scarcity of the studies on HPV in the conjunctiva, the variations in methods employed, and the interlaboratory variations for the same method, demonstrated in the literature, do not permit the establishment of an etiological relationship of HPV with conjunctival lesions. The present study leads us to suggest that some epithelial lesions of the conjunctiva have no association with the human papillomavirus.

## CONCLUSIONS

The low frequency of HPV found in the epithelial lesions of our sample and the detection of the viral genome in normal conjunctiva lead us to suggest that some conjunctival carcinomas have no association with the human papillomavirus. The etiopathogenic role of these viruses in the conjunctiva is yet to be established.
